# *Ex vivo* capillary-parenchymal arteriole approach to study brain pericyte physiology

**DOI:** 10.1117/1.NPh.9.3.031919

**Published:** 2022-06-23

**Authors:** Danielle A. Jeffrey, Jackson T. Fontaine, Fabrice Dabertrand

**Affiliations:** a University of Colorado Anschutz Medical Campus, Department of Anesthesiology, Aurora, Colorado, United States; b University of Colorado Anschutz Medical Campus, Department of Pharmacology, Aurora, Colorado, United States

**Keywords:** Brain microcirculation, pericyte, capillary, parenchymal arterioles, pressure myography

## Abstract

**Significance:**

Vascular mural cells, defined as smooth muscle cells (SMCs) and pericytes, influence brain microcirculation, but how they contribute is not fully understood. Most approaches used to investigate pericyte and capillary interactions include *ex vivo* retinal/slice preparations or *in vivo* two-photon microscopy. However, neither method adequately captures mural cell behavior without interfering neuronal tissue. Thus, there is a need to isolate vessels with their respective mural cells to study functional and pathological changes.

**Aim:**

The aim of our work was to implement an *ex vivo* method that recapitulates vessel dynamics in the brain.

**Approach:**

Expanding upon our established *ex vivo* capillary-parenchymal arteriole (CaPA) preparation, we isolated and pressurized arteriole-capillary branches. Using Alexa Fluor™ 633 Hydrazide, we distinguished arterioles (containing elastin) versus capillaries (lacking elastin). In addition, our transgenic SMMHC-GCaMP6f mice allowed for us to visualize mural cell morphology and ${\mathrm{Ca}}^{2+}$ signals. Lastly, isolated microvasculature was cultured in DMEM media (up to 72 h), mounted, and pressurized using our CaPA preparation.

**Results:**

U46619 induced a decrease in capillary lumen diameter using both a bath perfusion and local application. In addition, U46619 increased ${\mathrm{Ca}}^{2+}$ signaling both globally and locally in contractile pericytes. In our SMMHC-GCaMP6f mice, we saw that thin strand pericytes had sparse processes while contractile pericytes had long, thick processes that wrapped around the lumen of the capillary. Fresh and cultured pericytes constricted in response to U46619 to the same level, and upstream arteriolar dilation induced by capillary stimulation with 10 mM $K^{+}$ remained unchanged by culture conditions adding another application of longer treatment to our approach.

**Conclusion:**

Our *ex vivo* CaPA methodology facilitates observation of arteriolar SMC and pericyte dynamic changes in real-time without environmental factors. This method will help to better understand how mural cells differ based on microvasculature location.

## 1 Introduction

Vascular mural cells include smooth muscle cells (SMCs) and pericytes and have been shown to be important contractile units in the brain.^1^^–^^4^ Arteriolar ring-shaped SMCs cover larger vessels such as arteries and arterioles and are densely packed, while pericytes sit within the basement membrane of capillaries and have thin processes that run along the microvasculature.^5^^–^^8^ Mural cells are found throughout the body and, within the brain, are involved in normal vasculature formation such as angiogenesis, blood–brain barrier (BBB) integrity, and regulating cerebral blood flow (CBF).^9^^–^^11^ CBF regulation has recently become a topic of controversy, revolving around how or if pericytes orchestrate CBF regulation during functional hyperemia and mural cell participation during pathogenesis.^1^^,^^12^

Part of this controversy is due to the challenges in defining and studying mural cell types as their morphology dramatically changes based on microvasculature location, such as mural cells that associate with arterioles versus capillaries. This is particularly difficult when studying deep intracerebral (parenchymal) arterioles due to surrounding neuronal tissue as well as limitations in microscopy depth. Traditionally, studying microvasculature in the brain involves several techniques including *in vivo* two-photon imaging in the cerebral cortex,^3^ *ex vivo* retinal preparations,^13^^,^^14^ and cortical or cerebellar *ex vivo* slice imaging;^13^^,^^14^ however, limitations in these approaches exist. *In vivo* two-photon microscopy is limited by depth and dense surrounding neuronal tissue and thus acute focused treatment is limited in this methodology. *Ex vivo* retinal and brain slice preparations partially circumvent these issues with an increased experimental accessibility, but the presence of multiple cell types and the typical absence of luminal pressure can severely limit our interpretations.

To overcome these challenges presented by more traditional methods, we have created a preparation that focuses to remove microvasculature from its surrounding neuronal tissue where we can observe and apply pharmacological tools directly onto individual arterioles and capillaries and their respective mural cells. Our *ex vivo* capillary-parenchymal arteriole (CaPA) preparation enables us to remove microvasculature from hippocampal^15^^,^^16^ and cortical regions^17^^–^^21^ and elucidate changes in function. We have expanded upon our established method where we can treat vessels and mural cells acutely, either locally or globally via bath perfusion, or chronically from our cultured vasculature. Our approach can help to investigate mural cell function based on location such as SMCs on arterioles or pericyte subpopulations found on capillaries. This is advantageous as we can focus on how an individual cell is responding without the shielding response from other neuronal matter, i.e., neurons and astrocytes.

This method will provide a system to investigate individual mural cell behavior with the ability to stimulate a single pericyte and record functional changes such as lumen diameter changes or mural cell ${\mathrm{Ca}}^{2+}$ signaling. In addition, to add to the robustness of our preparation, we cultured isolated microvasculature in media for 48 to 72 h and proceeded with our CaPA preparation to measure lumen diameter changes post various pharmaceutical treatment and compared this to fresh isolated microvasculature from the same mouse. This addition opens the possibility for *ex vivo* extended exposure to various pharmaceutical or RNA interference reagents to measure functional changes. Overall, this method will help to better understand mural cell functional differences based on microvasculature location and give insight into how mural cells contribute to CBF regulation.

## 2 Materials and Methods

### 2.1 CaPA Preparation

**Fig. 1:**
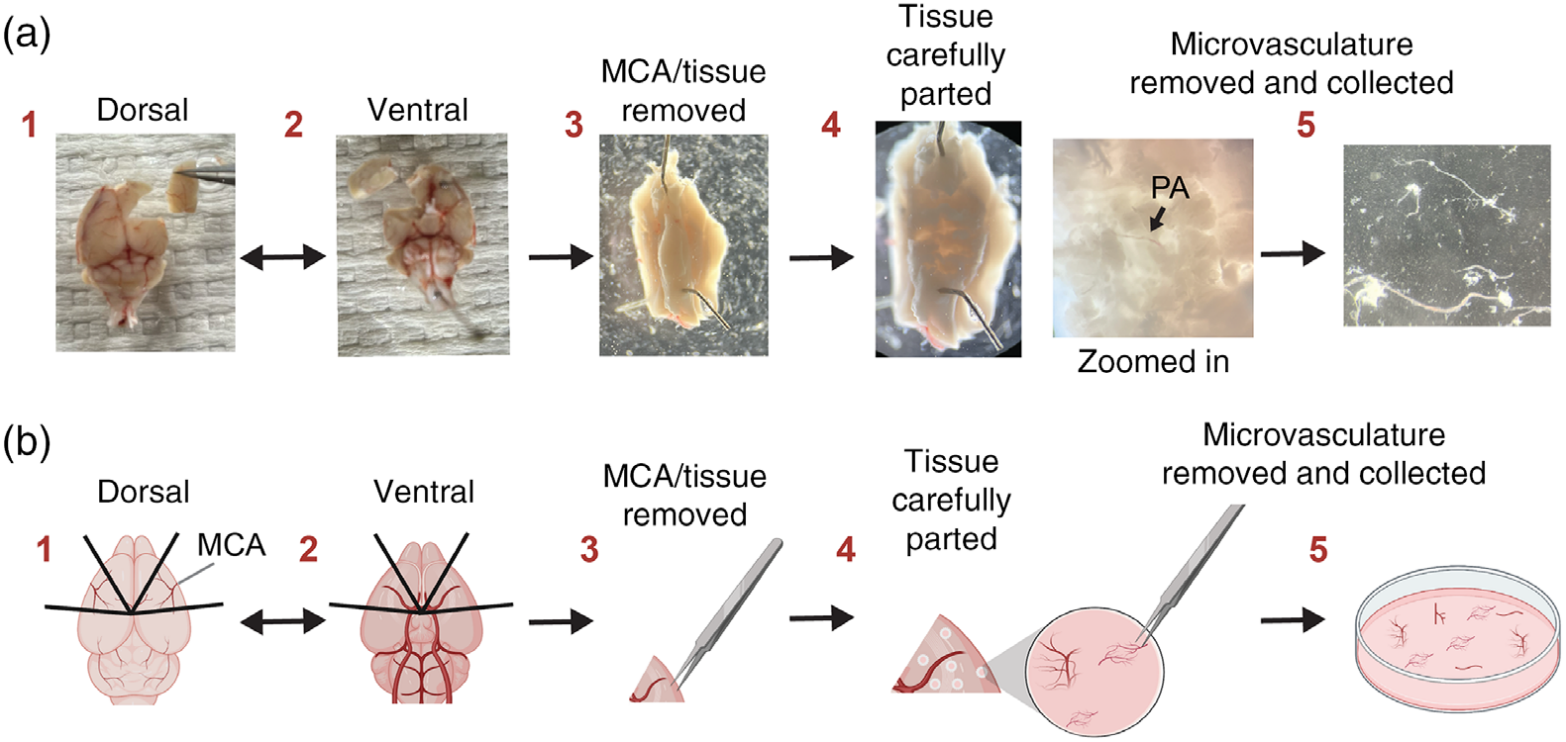
MCA dissection and isolated microvasculature workflow completed in MOPS. (a) Photographed images, (b) mirrored schematic diagram and workflow is as follows, 1: extracted mouse brain dorsal view with removed MCA section, 2: ventral view brain and MCA removed, 3: MCA tissue chunk isolated, 4: tissue very carefully and gently separated using forceps and zoomed-in vasculature is shown, 5: parenchymal arterioles and attached capillaries revealed, removed, and collected immediately followed by CaPA preparation. PA, parenchymal arteriole.

All animal experimental protocols were aligned with the institutional guidelines approved by the University of Colorado, Anschutz Medical Campus Institutional Animal Care and Use Committee. Mice were euthanized by intraperitoneal injection of sodium pentobarbital (100  mg/kg) followed by immediate decapitation. Cortical parenchymal arterioles were isolated as previously described.^15^^,^^16^ In short, the brain was removed after euthanasia and placed in 4°C MOPS-buffered saline (135 mM NaCl, 5 mM KCl, 1 mM ${\mathrm{KH}}_{2}{\mathrm{PO}}_{4}$, 1 mM ${\mathrm{MgSO}}_{4}$, 2.5 mM ${\mathrm{CaCl}}_{2}$, 5 mM glucose, 3 mM MOPS, 0.02 mM EDTA, 2 mM pyruvate, 10  mg/mL bovine serum albumin, pH 7.3 at 4°C). Under a dissecting scope, both sides of the middle cerebral artery (MCA) along with surrounding brain tissue were removed from the rest of the brain [Figs. 1(a) and 1(b)] while suspended in MOPS. This includes the M1—horizontal segment, M2—insular segment, M3—opercular segments (both superior and inferior trunks), and M4—cortical segment (both superior and inferior trunks). Notably other regions of the brain or vasculature may be selected, and the resulting methodology applied. Then using forceps and a ventral orientation secured by dissecting pins, the tissue was loosened by carefully putting the forceps into the tissue and gently opening them creating tiny perforations. This allowed for the visualization of parenchymal arterioles and corresponding capillaries. Then, parenchymal arterioles were carefully pulled out, dissecting them free of flanking neuronal matter. This gentle maneuver allowed for the capillaries to stay attached and undamaged. Isolated vasculature was then placed on ice in MOPS and those selected based on size and capillary branches were then cannulated.

Arterioles were cannulated with one end occluded and the ends of the capillaries were sealed by pressing them with a glass micropipette against the chamber coverslip as previously described.^15^ Application of luminal pressure (40 mmHg, estimated luminal pressure found *in vivo* for arterioles of this size^22^) applied to the cannulated arteriolar segment in this preparation pressurizes the entire vascular tree. The preparation was continuously perfused at 4  mL/min at 36.5°C (±1°C), with gassed (5% ${\mathrm{CO}}_{2}$, 20% $O_{2}$, and 75% $N_{2}$) artificial cerebrospinal fluid (aCSF; 125 mM NaCl, 3 mM KCl, 26 mM ${\mathrm{NaHCO}}_{3}$, 1.25 mM ${\mathrm{NaH}}_{2}{\mathrm{PO}}_{4}$, 1 mM ${\mathrm{MgCl}}_{2}$, 4 mM glucose, 2 mM ${\mathrm{CaCl}}_{2}$, pH 7.3) for at least 20 min prior to recording. Spontaneous constriction of the arteriolar segment induced by the luminal pressure, known as the myogenic response,^23^ was used as a marker for the preparation viability. Lumen diameter changes were continuously monitored using a charge-coupled device (CCD) camera and IonOptix software.

Pharmaceutical agents were applied either via bath perfusion or locally by precise pressure ejection with a Picospritzer^®^. Picospritzer^®^ cannula was placed ∼10 to 20  μm away from intended target. 100 nM U46619 (Tocris cat no. 1932), a thromboxane A2 receptor agonist (known pericyte constrictor^24^), was diluted in aCSF and gassed while perfused in the chamber bath in the same conditions as previously stated. The CaPA preparation was exposed to 100 nM U46619 in the bath for 3 min while lumen diameter changes were continuously monitored. The bath perfusion was only re-applied with a 10-min aCSF wash in between. To stimulate individual mural cells such as pericytes embedded in capillary walls, we used local pressure injection with a Picospritzer^®^ set to 20 s at a force of 5 pounds per square inch (psi). In addition, we added tetramethylrhodamine isothiocyanate (TRITC) (150 kDa) dextran to U46619-containing solutions and visualized the pericyte-capillary region stimulated by pressure ejection.

### 2.2 Transgenic Mice Utilization: SMMHC-GCaMP6f

Mural cells, including pericytes, express smooth muscle myosin heavy chain (SMMHC).^25^^,^^26^ We created a mouse line expressing the fast kinetics ${\mathrm{Ca}}^{2+}$ biosensor GCaMP6f expressed under the *Myh11* promoter. To avoid breeding complications from the biosensor being constitutively expressed, we bred C57BL6J Tg(Myh11-cre/ERT2)1Soff male mice expressing tamoxifen-inducible Cre under the control of the SMMHC (*Myh11*) promoter (The Jackson laboratory, stock no: 019079) with female mice Ai95D, which have a floxed-STOP cassette preventing transcription of the GCaMP6f indicator (The Jackson laboratory, stock no: 028865). Once mice reached 12 weeks, they were fed tamoxifen-containing chow for 2 weeks to allow for homozygous GCaMP6f expression. Our SMMHC-GCaMP6f mouse allowed us to visualize mural cell morphology and functional changes (i.e., ${\mathrm{Ca}}^{2+}$ signaling) using confocal and two-photon microscopy.

We utilized both female and male age-matched C57 mice (The Jackson Laboratory) when measuring lumen diameter changes for both fresh and cultured microvasculature CaPA preparations.

### 2.3 Alexa Fluor™ 633 Hydrazide Application to Distinguish Between Vasculature in Both *In Vivo* Imaging of CBF and *Ex Vivo* CaPA Preparation

Using retro-orbital injection,^27^ 1 to 2  mg/kg of Alexa Fluor™ 633 Hydrazide (Invitrogen, cat no. A30634) was administered to mice under isoflurane (5% induction, 2% maintenance) anesthesia. 60 min later, an acute cranial window surgery was implemented as previously described.^17^ Briefly, mice were anesthetized, and the skull was exposed followed by a circular cranial window drilled above the somatosensory cortex (roughly 2 mm in diameter). A stainless-steel head plate with a 2-mm diameter hole was then placed over the cranial window and secured with a mixture of dental cement and superglue. Isoflurane anesthesia was then replaced with α-chloralose (50  mg/kg) and urethane (750  mg/kg). Mice were kept at 37°C using an electric heating pad and monitored throughout surgery and recording with an anal probe thermometer. Utilizing SMMHC-GCaMP6f mice and two-photon laser-scanning microscopy (TPLSM), we then identified mural cells that fluoresced green under the *Myh11* promoter and distinguished arterioles versus capillaries based on Alexa Fluor™ 633 Hydrazide (red) uptake.

Separately, parenchymal arterioles with attached capillaries were isolated, *ex vivo* as previously described.^16^ Microvasculature was then incubated in 10  μg of Alexa Fluor™ 633 Hydrazide diluted in MOPS at room temperature for 20 min while rocking and covered to prevent light exposure. Microvasculature was then cannulated, pressurized, and perfused as described in the CaPA preparation (Sec. 2.1). Alexa Fluor™ 633 Hydrazide was then visualized using confocal microscopy ($\lambda_{\mathrm{Ex}}$: 640 nm; $\lambda_{\mathrm{Em}}$: 700 nm).

### 2.4 Confocal Microscopy: GCaMP6f ${\mathrm{Ca}}^{2+}$ Recordings

Post CaPA preparation, the vessel organ chamber was placed on a Nikon A1R Ti2 inverted confocal microscope using a CFI60 Plan Fluor 40× oil immersion objective lens (NA 1.3, WD 0.2 mm, FOV 25 mm, DIC). ${\mathrm{Ca}}^{2+}$ (GCaMP6f; $\lambda_{\mathrm{Ex}}$: 488 nm; $\lambda_{\mathrm{Em}}$: 525 nm) events were then captured by fast imaging (30 to 60 frames per second), via an ultrahigh-speed resonant scanner (7.8 kHz). Fluorescent changes in pericyte (soma + processes) or selected regions of interests (ROIs) were analyzed using a custom-designed noncommercially available SparkAn software (Dr. Adrian Bonev, University of Vermont).^28^^,^^29^ $F/F_{0}$ was normalized by taking baseline or basal ${\mathrm{Ca}}^{2+}$ fluorescence of 10 images. Vessels were stimulated with 100 nM U46619 with TRITC added to visualize ($\lambda_{\mathrm{Ex}}$: 640 nm; $\lambda_{\mathrm{Em}}$: 700 nm) local application of the drug.

### 2.5 IonOptix Edge-Detection Software and Lumen Calculations

For contractility and diameter studies, the organ chamber was placed on a Motic AE31 inverted microscope equipped with 10× (WD 16.8 mm) and 40× (WD 3 mm) objectives. Arteriolar or capillary lumen diameter was constantly monitored during perfusion and treatment using a CCD camera as previously described.^15^^,^^19^^,^^29^ Briefly, lumen diameter changes were acquired at 15 Hz in two regions using IonWizard 6.2 edge-detection software (IonOptix). Percent capillary lumen diameter changes were calculated using the following equation:

$$\frac{\left(\text{Diameter}\;{\left(\mu m\right)}_{\text{treated}}-\text{Diameter}\;{\left(\mu m\right)}_{\text{baseline}}\right)}{\text{Diameter}\;{\left(\mu m\right)}_{\text{baseline}}}*100.$$

### 2.6 Cultured Parenchymal Arterioles with Attached Capillaries

To add another application to the CaPA preparation, isolated cerebral parenchymal arterioles and capillaries were cultured. First, fresh isolated arteriole/capillary segments were mounted (CaPA preparation) and treated as described in Sec. 2.1. Other microvasculature from the same mouse and dissection were then cultured in 35 mm plates for 48 to 72 h in serum-free DMEM/F12 (Gibco, cat no. 11320-033) with 1% penicillin and streptomycin concentration and 2 mM glutamine in a Thermo Fisher 8000 incubator at 37°C with 5% ${\mathrm{CO}}_{2}$. To test viability, a pressurized CaPA preparation was performed as described in Sec. 2.1. 100 nM U46619 was locally applied using a Picospritzer^®^ (20 s at 5 psi), and lumen diameter changes were measured using IonOptix. Additionally in cultured CaPA microvasculature, 10 mM $K^{+}$ was locally applied using a Picospritzer^®^ (20 s at 5 psi) to capillaries and resulting arteriole lumen diameter was continuously recorded using edge-detection software (IonOptix). Percent change in lumen diameter was then determined and this was then compared to previously recorded fresh microvasculature from the same mouse dissection.

### 2.7 Image Processing, Analysis, and Statistics

*In vivo* TPLSM was captured on a Bruker Ultima Investigator multiphoton microscope using Prairie View software and a Spectra-Physics Mai Tai^®^ eHP DeepSee™ ultrafast laser. The system integrates two imaging channels with high sensitivity GaASP photomultiplier tubes. Excitation was done at 810 nm and green and red fluorescence emission were collected through 525/70-nm and 595/50-nm bandpass filters.

*Ex vivo* microvascular lumen diameter imaging and lumen diameter values were acquired using IonOptix software from a CCD camera. Representative traces were configured in GraphPad Prism 9. *Ex vivo* confocal microscopy was performed using a Nikon A1R confocal and NIS-Elements AR 5.21.03 version software. Z-series captured on the Nikon Eclipse Ti2 with 0.25  μm step sizes were used to capture the entire three-dimensional microvasculature structure.

All statistical tests were performed in GraphPad Prism 9. All data were checked for normality (skewness and frequency histograms were observed) and if normally distributed a paired (all dependent data or microvasculature from the same mouse) or unpaired (all independent data or microvasculature from different mice) t-test was used. Significance was assigned as α<0.05 and error bars denote ± SEM. Each corresponding statistical test is denoted in the figure legend.

## 3 Results and Discussion

### 3.1 CaPA Preparation Facilitation

Brain circulation has been studied for decades, yet with less emphasis on parenchymal arterioles and their respective capillary network. Recently, more attention has been brought to pericytes and their role in orchestrating CBF in both healthy and diseased brains.^25^^,^^30^ This attention has revealed that pericytes are not passive bystanders in the brain microenvironment but help to orchestrate CBF during functional hyperemia.^17^^,^^30^^–^^32^ In addition, the pericyte’s role in neurodegenerative diseases is of growing interest^3^^,^^15^^,^^30^^,^^33^^,^^34^ and understanding this heterogeneous population may be the key to providing new therapies to combat neurodegenerative disease progression. Typical approaches to study pericyte function include *in vivo* two-photon microscopy,^25^ *ex vivo* retinal preparations,^4^ and *ex vivo* brain slices.^13^^,^^14^ We propose a unique method that enables the observation of brain capillaries and associated pericytes (both contractile and thin-strand), without surrounding neuronal tissue. This allows for experiments to be conducted to test dynamic pericyte function in real-time and how pericytes affect their corresponding microvasculature.

**Fig. 2:**
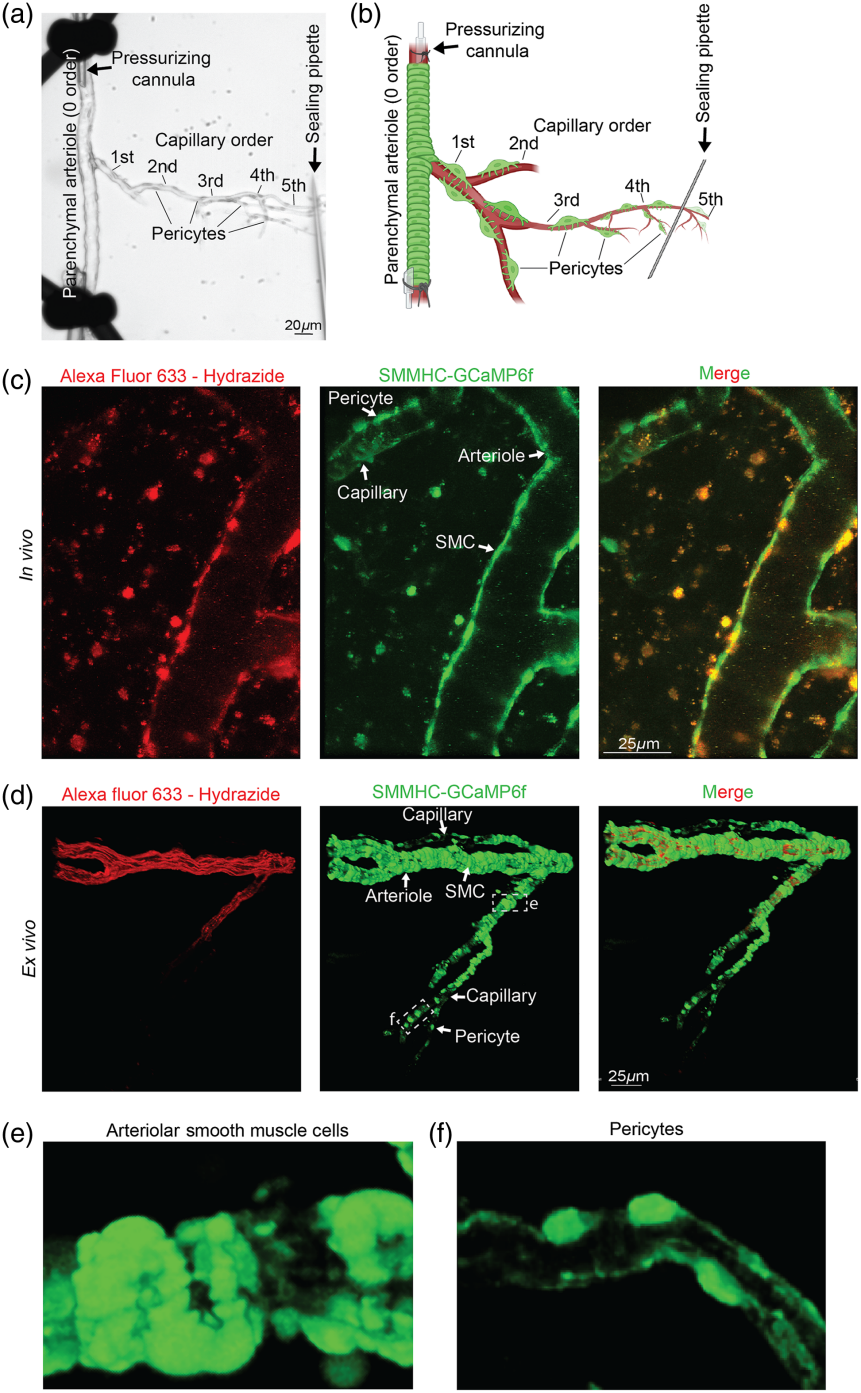
Unbiased capillary/parenchymal arteriole distinction using elastic lamina marker Alexa Fluor™ 633 Hydrazide. (a) CaPA preparation set up of microvasculature isolated from middle cerebral arterial cortex region. (b) Schematic representation of a CaPA preparation created with BioRender.com. (c) *In vivo* TPLSM (Bruker Ultima Investigator multiphoton microscope coupled to a Spectra-Physics Mai Tai^®^ eHP DeepSee™ ultrafast laser) images of microvasculature post Alexa Fluor™ 633 Hydrazide retro-orbital injection and cranial window surgery. Representative image is a snapshot from a T-series. SMC, smooth muscle cell. (d) *Ex vivo* CaPA preparation post Alexa Fluor™ 633 Hydrazide incubation using Nikon A1R confocal microscopy. Representative image is a maximum intensity projection from a Z-stack (0.25  μm steps and deconvoluted). Boxes in middle panel represent zoomed in image of mural cell morphology for (e), (f) (c), (d) Channels: red – Alexa Fluor™ 633 Hydrazide, green – GCaMP6f, and merged channels. Microvasculature structures are denoted by arrows and scale bar is shown. (e) Close-up of SMC morphology from SMMHC-GCaMP6f only channel. (f) Close-up of pericyte morphology from SMMHC-GCaMP6f only channel.

Figure 2(a) is a brightfield image of our CaPA preparation. Parenchymal arterioles with attached capillaries were isolated as described. The arteriole is then cannulated and tied (pressurized cannula) while the other end of the arteriole is tied off. The attached capillary branches are then spread and pinned with the sealing pipette. The arteriole is then continuously perfused with aCSF and luminally pressurized at 40 mmHg, and this creates a closed pressurized system that recapitulates the environment within the brain (36.5°C gassed with 5% ${\mathrm{CO}}_{2}$, 20% $O_{2}$, and 75% $N_{2}$). Figure 2(a) displays how this method enables us to observe and interrogate more than one capillary or pericyte at a time, which can be challenging *in vivo*. In addition, we find that denoting branch order *in vivo* can be challenging due to the vast capillary network that exists within the brain, therefore our *ex vivo* CaPA preparation allows for a clear distinction in vasculature branching [Figs. 2(a) and 2(b)]. Figure 2(b) shows a schematic which represents how the sealing of the capillary branch can be adjusted to capillary order such as prior to thin strand pericytes (typically found on higher order capillaries^35^) to focus on contractile pericytes [Fig. 2(b)] or after higher order capillaries to observe thin strand pericytes [Fig. 2(a)]. This is another advantage of our CaPA preparation, which allows for the control of pressurized capillary branches, where based on the location of the sealing pipette we can observe functional changes in isolated pericyte subpopulations.

### 3.2 *Ex vivo* CaPA vs *In Vivo:* Alexa Fluor™ 633 Hydrazide

Previous studies have established an artery-specific fluorescent dye, Alexa Fluor™ 633 Hydrazide, that differentiates between arterioles and capillaries in the brain.^36^^,^^37^ Alexa Fluor™ 633 Hydrazide binds selectively to elastin, which is present in arterioles however absent in capillaries.^36^^,^^37^ To investigate this *in vivo* in our SMMHC-GCaMP6f mice, Alexa Fluor™ 633 Hydrazide was injected retro-orbitally.^27^ Using TPLSM, the observed fluorescent staining distinguished arterioles versus capillaries based on the protocol published by Shen et al.^36^ We were able to detect red fluorescence indicative of arterioles while capillaries did not contain Alexa Fluor™ 633 Hydrazide in their walls [Fig. 2(c)]. However, in addition to arterioles we noticed potential veins and elastic fibers taking up more fluorophore, respectively (Fig. S2 in the Supplemental Material). This could be because we applied the Alexa Fluor™ 633 Hydrazide via retro-orbital injection, where Shen et al. delivered it by intravenous (tail) injection. However, other fluorophores such as TRITC and FITC in our laboratory are also delivered this way, and we do not see external vasculature fluorescence. While both methods of venous delivery have resulted in similar efficacy, the retro-orbital technique has been found to be faster,^38^ and we can only speculate that this may contribute to our differences in staining from Shen et al. They also noticed probe fluorescence over 96 h post-injection;^36^ however, we found that 3 h later fluorescence was fading.

Interestingly, in our *ex vivo* CaPA preparations when we incubated vessels in Alexa Fluor™ 633 Hydrazine, we were able to clearly distinguish between arteriole (containing elastin) and capillary (lacking elastin) segments using confocal microscopy. Alexa Fluor™ 633 Hydrazine was present in the arteriole walls yet faded in transitional segments to indistinguishable in the capillary branches [Fig. 2(d)]. This provides another degree of certainty when characterizing vasculature in addition to the lumen diameter and mural cell morphological properties that is useful in our *ex vivo* CaPA methodology. This is highlighted in Figs. 2(e) and 2(f) where SMC and pericyte morphology are visualized using our CaPA methodology.

### 3.3 *Ex Vivo* CaPA U46619 Treatment: Local Application Versus Bath Perfusion

**Fig. 3:**
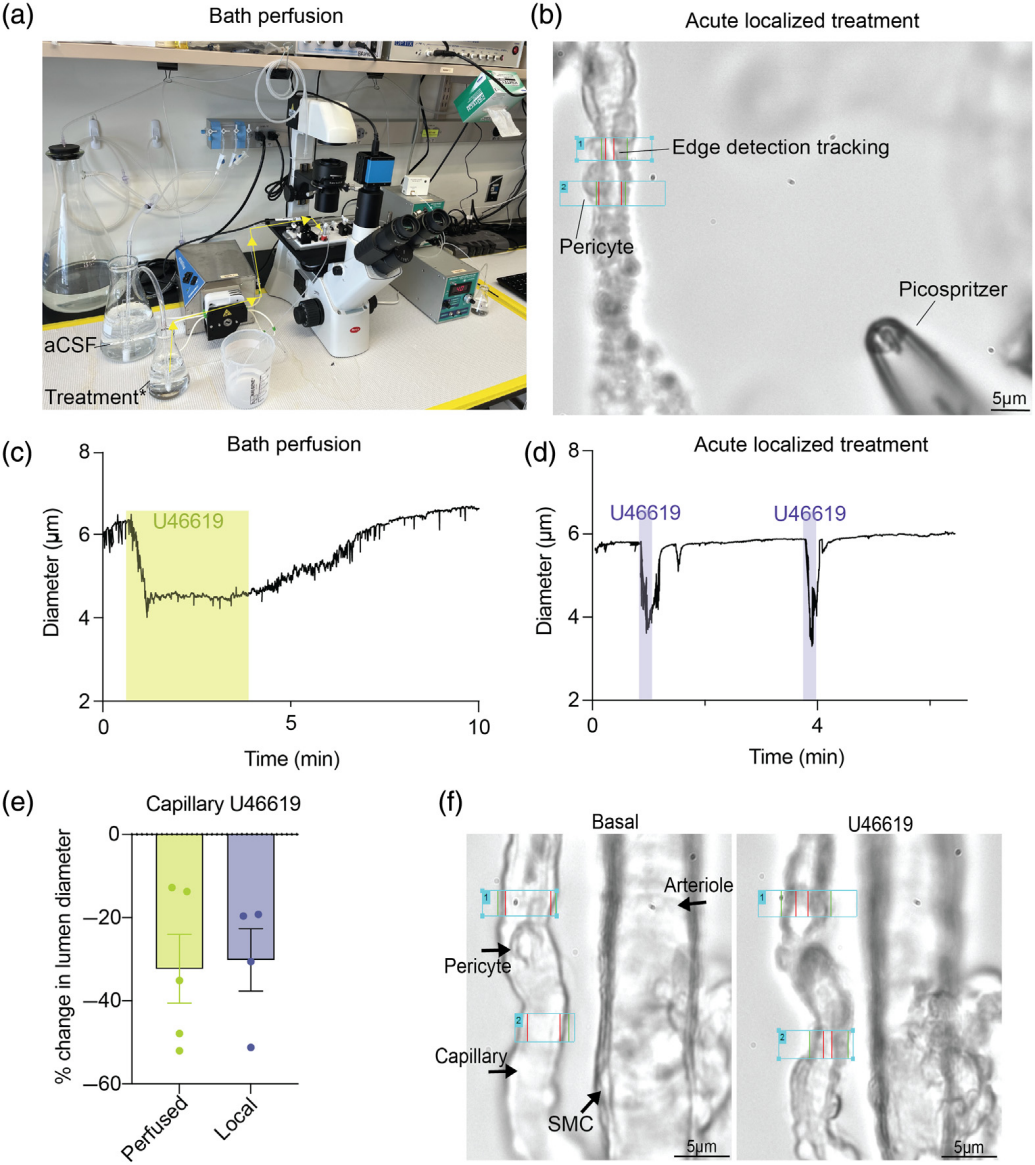
Perfused versus local U46619-application post *ex vivo* CaPA preparation. (a) Bath perfusion set-up apparatus. *Yellow arrows indicate treatment flow to vasculature. aCSF, artificial cerebral spinal fluid. (b) CCD camera brightfield image from IonOptix software showing precise localized treatment using a *pipette connected to the pressure ejection device (Picospritzer^®^). Scale bar is shown, and labels are indicated. (c) Representative trace of pressurized (40 mmHg) isolated capillary luminal diameter upon 100 nM U46619-induced constriction via bath perfusion for 3 min. (d) Representative trace of pressurized (40 mmHg) isolated capillary upon 100 nM U46619-induced constriction using a Picospritzer^®^ (20 s, 5 psi), two consecutive treatments are shown. (e) Quantification of percent change in lumen diameter in capillaries for bath perfusion versus local (Picospritzer^®^) application. Error bar denotes ± SEM, n=5 for bath perfusion and n=4 for local application. Unpaired t-test revealed not significant (P=0.86). (f) CCD camera brightfield image from IonOptix software showing microvasculature in basal conditions and after U46619 bath application to show vasoconstriction upon treatment. Scale bar is shown, and microvasculature is labeled.

U46619, a thromboxane A2 receptor agonist, is a known pericyte constrictor and has been shown to constrict cerebral capillaries *in vivo*.^4^^,^^24^ To confirm the potency of this treatment in our *ex vivo* CaPA preparation, we used two different treatment applications and sought to compare how capillaries respond to either continuous bath perfusion versus local focused treatment. Figure 3(a) shows a diagram showing our bath perfusion set up. The aCSF and treatment (diluted in aCSF) is preheated (36.5°C) and continuously bubbled as previously described. Figure 3(a) shows the path of the bath perfusion treatment (indicated by yellow arrows) which then enters the bath, and the microvasculature is exposed externally. Figure 3(b) shows a brightfield image of our CaPA preparation focusing on a single capillary and how we can precisely deliver treatment via a pipette attached to pressurized Picospritzer^®^ apparatus and continuously monitor lumen diameter changes (edge-detection software, IonOptix).

Focusing on this dual-treatment approach using our CaPA methodology, we found that in the bath perfusion treatment, the capillary response to U46619 was much more gradual and maximal constriction took longer to occur [Fig. 3(c)]. However, with the localized treatment (Picospritzer^®^), a quick capillary constriction in response to 100 nM U46619 occurred [Fig. 3(d)]. In addition, we noted that localized treatment enabled us to treat the vasculature twice within a 5-min period with a recovery period in between [Fig. 3(d)]. This difference in response is likely due to the dilution of the bath perfusion to reach full concentration, while the local application delivers 100 nM U46619 instantly to the pericyte-capillary segment, resulting in a faster vasoconstriction. This is shown in Fig. S3 in the Supplemental Material. Reciprocally, return to baseline diameter after U46619 treatment took longer via batch perfusion compared to Picospritzer^®^, likely due to the slower clearance.

While the bath perfusion induced a slower constriction, there was no significant difference in percent change in capillary lumen diameter in the localized (mean constriction = 32.31%) versus bath perfusion (mean constriction = 30.16%) delivery of 100 nM U46619 [unpaired t-test P value = 0.86; Fig. 3(e)]. To appreciate the vasoconstriction seen at the capillary level, Fig. 3(f) shows a CCD image of a contractile pericyte and respective capillary both at basal conditions and 3 min of 100 nM U46619 bath application where a noticeable capillary lumen diameter decrease is seen. While both localized and bath application approaches have their benefits, as shown in Fig. 3(f), the bath application is not specific and results in the entire microvasculature unit being stimulated, which can be advantageous in certain scenarios. The local delivery of the agonist offers better control over the stimulation window, which can be useful in certain studies of the on/off kinetics. However, the major benefit remains the ability to stimulate one site rather than the entire preparation.

### 3.4 Mural Cell Morphology Characterization Using Confocal Microscopy

Mural cell morphology dramatically changes based on microvasculature location. Furthermore, many have found that mural cell markers (such as PDGRβ or NG2) change throughout the microvasculature where branch order does not always align with expression.^1^^,^^12^^,^^39^ Previous studies have described SMCs located on larger vessels such as arterioles as having ring-shaped cells while pericytes have a more bump-on-a-log shape with processes that run along the capillary walls.^5^ The pericyte population is heterogeneous in nature where capillary order appears to dictate pericyte morphology and expression^1^^,^^5^^,^^10^^,^^14^ although not absolute,^39^ which makes them difficult to study due to their location and seemingly several subtypes. Our CaPA methodology is unique in that we can isolate microvasculature and pin individual capillaries to observe individual mural cells such as contractile pericytes located on first- to fourth-order capillaries and compare that subset to thin-strand pericytes typically located on ≥ fifth-order capillaries. Using our SMMHC-GCaMP6f mouse model, we were able to selectively observe mural cells by confocal microscopy with high spatial resolution to characterize their morphology using our CaPA preparation.

**Fig. 4:**
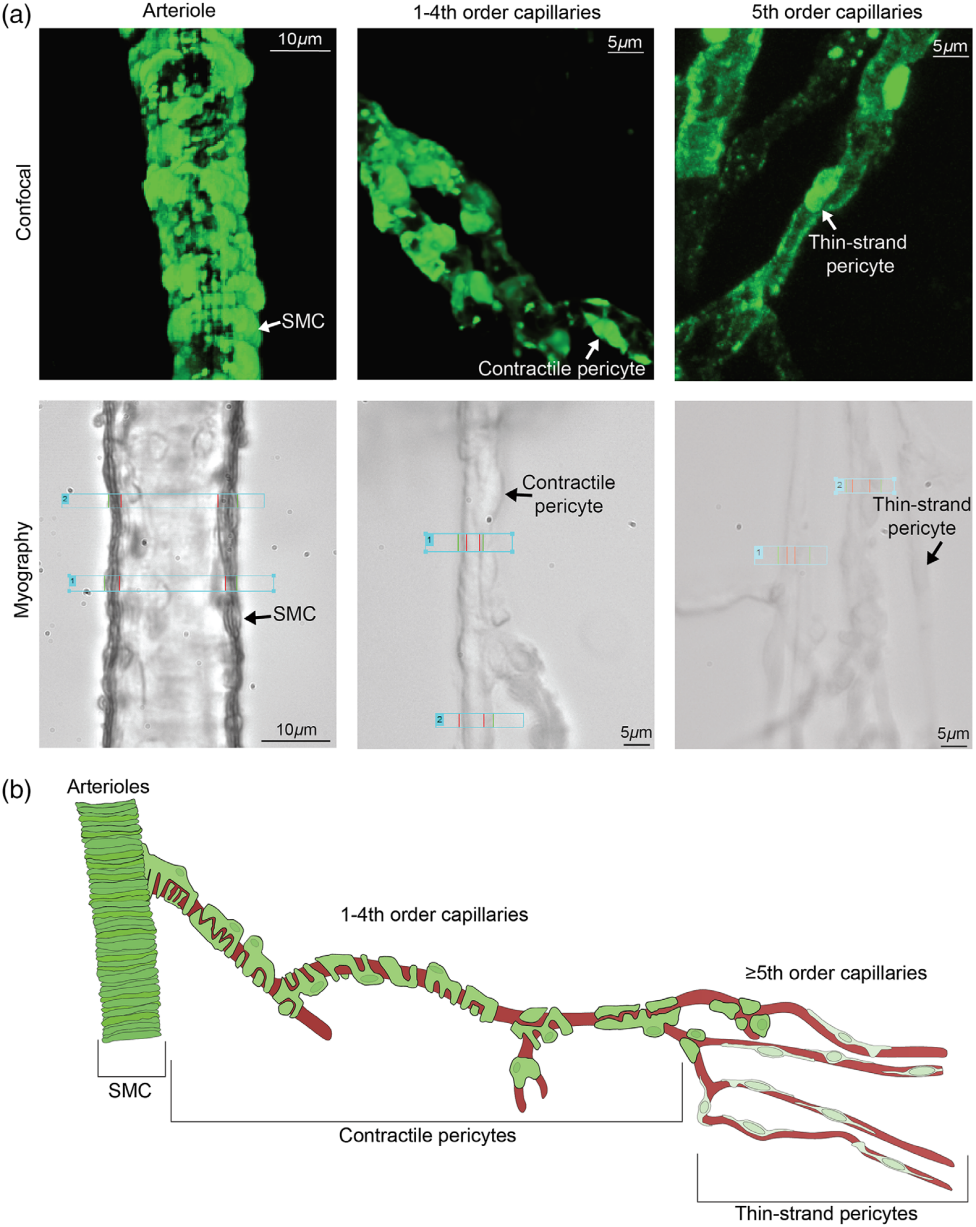
Morphology characterization of mural cells post *ex vivo* CaPA preparation. Top panel: Confocal imaging on CaPA preparation mural cell morphology shown in green (SMMHC-GCaM6f). Representative image is a maximum intensity projection from a Z-stack (0.25  μm steps, deconvoluted). Bottom panel: CCD camera myography brightfield imaging of CaPA preparation. Scale bar is shown in both. (a) Left panels: Arteriolar SMCs on arterioles with more ring-like structures. Lateral sides of rings are seen in the myography image. Middle panels: Contractile pericytes typically seen on first- to fourth-order capillaries. Round body is seen with dense projections that follow the lumen of the capillary. The side projection of the contractile pericyte body can be clearly seen in the myography panel. Right panels: Thin strand pericytes typically found on ≥ fifth-order capillaries. Shallow pericyte body with thin-sparse processes can be seen in both the confocal and myography images. (b) Representative schematic of mural cell morphology based on microvasculature location.

We found SMCs had ring-like shaped cells that encircled the endothelium and were located on larger vasculature such as arterioles [Fig. 4(a)]. Transitioning to capillaries, however, we found that the contractile pericytes had large bump-like somas with an outward protruding nucleus and dense processes that wrapped around or ran longitudinally with the vessel lumen [Fig. 4(a)]. In addition, when we reached higher branched capillaries, we found that thin-strand pericytes looked more like distal noncontractile pericytes and had a shallow nucleus with sparse processes that ran longitudinally along the vessel lumen and had some processes that wrapped around endothelial cells [Fig. 4(a)]. In addition, using our *ex vivo* CaPA myography setup, we also visualized mural cells while recording lumen diameter changes. Brightfield microscopy images revealed the same findings as shown in Figs. 2(e) and 2(f) and Fig. 4(a). SMCs had many ring-like cell structures in close proximity with one another whereas contractile pericytes had a protruding nucleus with dense processes [Fig. 4(a)]. In addition, thin-strand pericytes found in higher order capillaries had a flat nucleus without noticeable processes encircling the corresponding endothelial cells [Fig. 4(a)].

Based on these observations, Fig. 4(b) shows a schematic cartoon of how mural cell morphology changes based on location. In arterioles, we show densely packed SMCs that encircle the vessel lumen. As the vasculature transitions to first- to fourth-order capillaries, the contractile pericytes have bump-like nuclei with close-knit processes that run along and encompass the lumen walls. In this region, mural cells fell under the mesh, rather than ensheathing, pericyte subcategory described by Grant et al.^5^ Finally, in higher order capillaries (≥ fifth-order), the pericyte subpopulation then changes to thin-strand pericytes, which have shallow nuclei and shorter, more sporadic processes that encircle and run along the capillary walls. It is important to note that we appreciate the heterogeneity of the pericyte population and acknowledge that branching order and corresponding pericyte subtype is not always concrete.^39^ In support of that, Fig. 2(d) shows a pure capillary (no Alexa Fluor™ 633) branching from the cannulated arteriole whereas the inferior branch contains elastin and has a thicker wall (brightfield image, Fig. S1 in the Supplemental Material) which would indicate it is an arteriole. Therefore, branch order (first for both) would not be appropriate in defining the vasculature type or corresponding mural cell. Shaw et al. emphasized that gradual transitions can occur throughout the microvasculature and even branch bifurcations have specialized mural cells^39^ adding another layer of complexity. Therefore, our *ex vivo* CaPA preparation is advantageous as branching order can be clearly distinguished while utilizing brightfield, fluorescence, and expression interrogation when we isolate microvasculature, which is not as easily appreciated using *in vivo* cranial window modeling or *ex vivo* brain slices.

### 3.5 *Ex Vivo* CaPA ${\mathrm{Ca}}^{2+}$ Signaling in Contractile Pericytes

${\mathrm{Ca}}^{2+}$ signaling and corresponding contractility regulation has been well defined in arteriolar SMCs, however, less is known about pericytes and their respective capillaries.^31^ To add to the complexity of cerebral microvasculature, pial versus parenchymal arterioles display different responses to luminal pressure and, throughout the vascular tree, their ${\mathrm{Ca}}^{2+}$ signals also differ.^29^^,^^40^^–^^42^ Therefore, due to the differences in cerebral arteriole ${\mathrm{Ca}}^{2+}$ signaling, one would infer there are differences in pericyte subpopulations as well. Glück et al. shown *in vivo* (TPLSM) and *ex vivo* (brain slices) that capillary pericytes displayed spontaneous localized ${\mathrm{Ca}}^{2+}$ events that were frequent and irregular, in both the pericyte soma and processes in response to U46619.^6^ During neurovascular coupling, Rungta et al. also recorded different kinetics in ${\mathrm{Ca}}^{2+}$ signals between mesh and thin strand pericytes.^42^ We therefore tested the ability of our approach to capture pericyte ${\mathrm{Ca}}^{2+}$ signaling using our CaPA preparation in conjunction with the SMMHC-GCaMP6f mice.

**Fig. 5:**
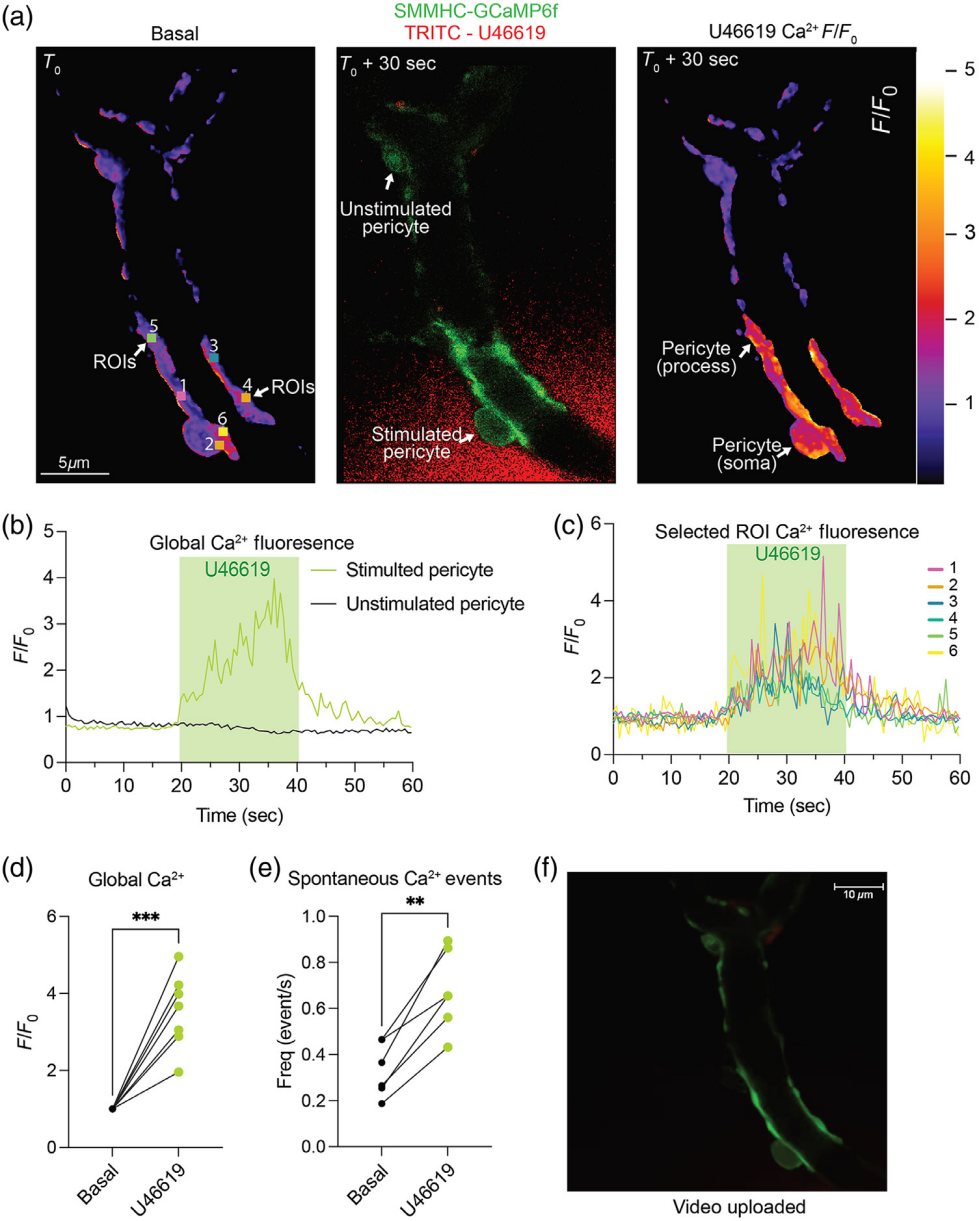
*Ex vivo* CaPA preparation ${\mathrm{Ca}}^{2+}$ imaging upon U46619 stimulation. (a) Fluorescent intensity imaging (GCaMP6f) of basal pericyte ($T_{0}$; left panel) and during TRITC-U46619 acute localized exposure ($T_{0}+30\;s$; central and right panels) by pressure ejection (Picospritzer^®^). Selected ROIs are displayed in basal (left) panel. TRITC (red) fluorescence indicative of U46619 reaching a singular pericyte/capillary in middle panel. Intensity of GCaMP6f fluorescence is shown as $F/F_{0}$ in the right panel with corresponding scale bar. (b) Global ${\mathrm{Ca}}^{2+}$ representative trace ($F/F_{0}$ intensity change) upon local 100 nM U46619 treatment. Duration and location of treatment is noted, Picospritzer^®^ (20 seconds, 5 psi, ∼15  μm away). (c) Representative trace of ROIs that were selected around the pericyte soma and along the processes. Duration and location of U46619 treatment is noted, Picospritzer^®^ (20 s, 5 psi). Color-coded legend matches selected ROIs from (a). (d) Quantification of global ${\mathrm{Ca}}^{2+}$ fluorescence signaling during 100 nM U46619 treatment. N=7 independent CaPA preparations (*** indicates P<0.001, P=0.0005, paired t-test). (e) Spontaneous ${\mathrm{Ca}}^{2+}$ event frequency, n=5 independent CaPA preparations (** indicates significance P<0.01, P=0.001, paired t-test). (f) Video 1, individual pericyte perturbed by 100 nM U46619 indicated by TRITC (red) fluorescence and resulting ${\mathrm{Ca}}^{2+}$ signaling (GCaMP6f). Scale bar and time scale is indicated. Red gain was adjusted (lowered) to appreciate GCaMP6f fluorescence, resulting in TRITC appearing slightly after the beginning of the pericyte response (Video 1, MP4, 218 KB [URL: https://doi.org/10.1117/1.NPh.9.3.031919.1]).

Using confocal microscopy, we imaged contractile pericytes located on first- to fourth-order capillaries. We recorded GCaMP6f-emitted fluorescence during basal conditions to obtain a baseline and then locally treated (Picospritzer^®^, 20 s, 5 psi, ∼15  μm away) a single pericyte with 100 nM U46619 aCSF and TRITC [Fig. 5(a)] to visualize U46619 delivery. ${\mathrm{Ca}}^{2+}$ signaling was then recorded and quantified ($F/F_{0}$) using SparkAn software.^28^^,^^29^ U46619 caused an increase in global ${\mathrm{Ca}}^{2+}$ signaling using local simulation [Fig. 5(b)]. We also quantified ${\mathrm{Ca}}^{2+}$ signaling in the unstimulated pericyte [Fig. 5(a)] and found that global ${\mathrm{Ca}}^{2+}$ fluorescence did not increase upon localized U46619 treatment [Fig. 5(b)]. In addition, using selected ROIs [Fig. 5(a)] there was an increase in ${\mathrm{Ca}}^{2+}$ fluorescence in both the pericyte soma and processes [Fig. 5(b)], similar to what Glück et al. reported. Figure 5(c) shows a representative trace of selected ROIs [in Fig. 5(a)] with an increase of $F/F_{0}$ during localized U46619 stimulation. We then quantified global ${\mathrm{Ca}}^{2+}$ events in seven different independent CaPA preparations and found a significant increase in U46619-induced ${\mathrm{Ca}}^{2+}$ global events [Fig. 5(d)] when compared to fluorescent baselines (paired t-test P=0.0005). We also observed a significant increase in spontaneous ${\mathrm{Ca}}^{2+}$ events [Fig. 5(e)] during localized U46619 treatment (n=5, paired t-test P=0.001). Figure 5(f) shows a time stamped video of a contractile pericyte being stimulated with U46619 (TRITC-red, added for visualization) and resulting ${\mathrm{Ca}}^{2+}$ SMMHC-GCaMP6f (green) fluorescence. Note how the unstimulated pericyte does not respond (have any increase in ${\mathrm{Ca}}^{2+}$ green fluorescence).

These results compound upon previous outputs from other groups and further add to the validity of our *ex vivo* CaPA preparation to study functional changes at the pericyte level. Parenchymal arterioles are difficult to study along with the elusive heterogenous pericyte subpopulation, yet this method helps to isolate these cells and stimulate them individually and observe/measure functional changes. This methodology will help to quantify functional ${\mathrm{Ca}}^{2+}$ signaling in different subpopulations of pericytes highlighting potential unknown differences.

### 3.6 Cultured Cerebral Microvasculature and Resulting CaPA Preparation

**Fig. 6:**
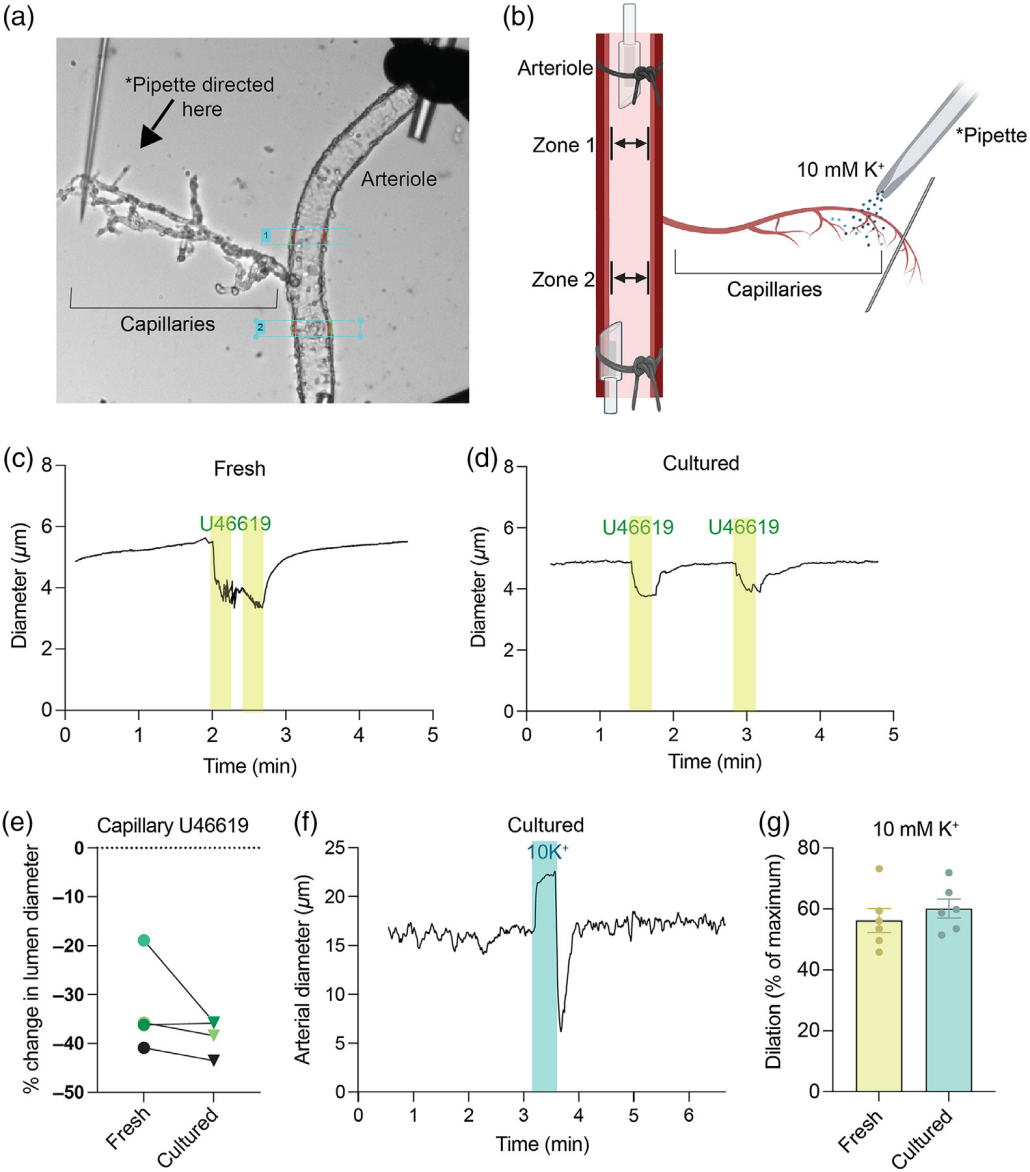
Fresh versus cultured *ex vivo* CaPA preparation. (a) CCD camera brightfield image from IonOptix software of a 48-h cultured vessel and resulting CaPA preparation. (b) Schematic diagram of 10 mM $K^{+}$ localized treatment created with BioRender.com. Arteriole and capillary segments were mounted 48 h post culture. *Pipette connected to the pressure ejection device (Picospritzer^®^) where capillaries were stimulated while simultaneously downstream arteriole lumen diameter changes were recorded. (c) Representative trace of lumen diameter changes of a freshly isolated capillary at a contractile pericyte location showing the effect of U46619 locally applied. Duration of U46619 application is indicated, Picospritzer^®^ (20 s, 5 psi). (d) Representative trace of lumen diameter changes of a cultured (48 h) capillary at contractile pericyte location showing the effect of U46619 locally applied. Two consecutive U46619 treatments were applied as indicated, Picospritzer^®^ (20 s, 5 psi). (e) Quantification of percent change in lumen diameter in fresh versus cultured capillaries. Lines connecting points indicate four independent paired microvasculature isolations. Paired t-test not significant (P=0.2519). (f) Representative trace of 10 mM $K^{+}$ locally applied to capillaries and measured downstream changes in arteriole diameter. Duration of treatment is indicated, Picospritzer^®^ (20 s, 5 psi). (g) Quantification of arteriole dilation (percent of maximum) of fresh (n=6) versus cultured (n=6) arterioles. Error bars denote ± SEM, unpaired t-test (fresh versus cultured vasculature was isolated from different mice) p-value not significant (P=0.45).

While we have shown that acute treatment is viable using our CaPA preparation, we wanted to investigate cultured microvasculature to add a chronic treatment application to our methodology. Using extracted microvasculature from the cerebral cortex we cultured the vessels in serum-free DMEM + 2 mM glutamine for 48 to 72 h. Figure 6(a) shows a brightfield image of a 48-h cultured CaPA preparation whereas Fig. 6(b) shows a schematic diagram to illustration the application of $K^{+}$ to capillaries while recording upstream arteriole lumen diameter changes. To demonstrate that the vasculature could be cultured and respond such as fresh isolated microvasculature, we mirrored the same experiments as performed previously. 48-h cultured capillaries were locally stimulated using a Picospritzer^®^ while mounted (CaPA preparation). Capillaries constricted in response to 100 nM U46619 treatment as seen in fresh preparations [Fig. 6(c)]. Additionally, in microvasculature from the same mouse, our cultured capillaries also constricted in response to U46619 followed by a return to baseline and constricted when stimulated again [Fig. 6(d)]. We repeated this method with four independent mice over four different days and found that there was no significant difference in the U46619 response [Fig. 6(e)] in fresh (mean constriction = 32.92%) versus cultured (mean constriction = 38.36%) capillaries according to a paired t-test (P=0.25).

Previously, we have shown that 10 mM $K^{+}$ localized treatment to capillaries results in an upstream dilation of the arteriole segment.^17^^–^^20^ To ensure that these cultured vessels responded in the same way, we repeated this treatment on 48-h cultured microvasculature. Figure 6(b) shows a schematic diagram of our experimental apparatus. The microvasculature was mounted as previously described and the edge detection software (IonOptix) zones 1 and 2 were focused on the arteriole lumen while the capillaries are stimulated. As previously described,^17^ we saw that in response to localized capillary 10 mM $K^{+}$ treatment, the arteriole dilated followed by a fast constriction [Fig. 6(f)]. We then repeated this (fresh n=6 and cultured n=6) and saw there was no significant difference (P=0.45, unpaired t-test) in fresh (mean dilation = 56.23%) versus cultured (mean dilation = 60.12%) arteriole-capillary segments [Fig. 6(g)].

These data support the notion that our *ex vivo* CaPA preparation is robust with both acute and chronic application. The 48-h cultured vessels are still viable and respond the same as fresh microvasculature confirming that this method can be used to expose vessels to long-term treatment and then functionally observed.

### 3.7 Limitations

We have introduced a method with diverse applications, yet we acknowledge that there are limitations to this approach. (1) While it can be beneficial to interrogate pericyte function without surrounding neuronal matter, this will not precisely mimic stimuli that would occur *in vivo*. Isolating vasculature without their neighboring cell types allows for more control over what we are observing; however, we do lose several of the cell types that compose the neurovascular unit. Hence, we encourage validating findings *in vivo.* (2) While our CaPA preparation provides pressurized vasculature, in higher order capillary branches it is not always feasible to pin every branch. These capillary branches remain pressurized due to upstream pressure, but we admit it is not always a completely closed system. To address this issue, in our recordings we only measure capillaries that have a sealed end. (3) While this method can help to elucidate differences between the pericyte subpopulations, we are also removing the microvasculature from its complete network (larger upstream arterioles/arteries or downstream venules/veins), and we acknowledge this as a limitation and again suggest supplementing observations with *in vivo* studies. (4) Culture conditions described here affected neither the U46619-induced constriction nor the electrical integrity of the preparation, but other features such as the BBB may be different. Specially, ensuring normoxia in incubators can be challenging, and longer culture durations would certainly impair the preparation viability.

## 4 Conclusion

Our developed and expanded upon *ex vivo* CaPA preparation is a reliable model that recapitulates the microvasculature workings in the brain. We have demonstrated that this method is ideal for monitoring pericyte-capillary functional changes, especially on higher order capillary segments, which can be challenging to study using other approaches. In addition, we characterized mural cell morphology utilizing our SMMHC-GCaMP6f mouse model in thin-strand pericytes located on higher-order capillaries, contractile pericytes typically located first- to fourth-order capillaries, and SMCs located on arterioles. We then perturbed them individually with U46619 which resulted in capillary constriction. This approach also works with cultured (48 to 72 h) microvasculature enabling chronic versus acute exposure, which adds to the functionality and robustness of this preparation. Importantly, these techniques can be utilized to better understand how mural cells contribute to orchestrating cerebral blood flow or participate in pathogenesis and give insight into this elusive cell population.
