# Supplementary material for: Ex vivo capillary-parenchymal arteriole approach to study brain pericyte physiology
Source: Neurophotonics. 2022 Jun 23;9(3):031919. doi: 10.1117/1.NPh.9.3.031919 (PMC9225307; doi:10.1117/1.NPh.9.3.031919)
Supplement: Supplementary file 1 [file NPh_009_031919_SD001.docx]

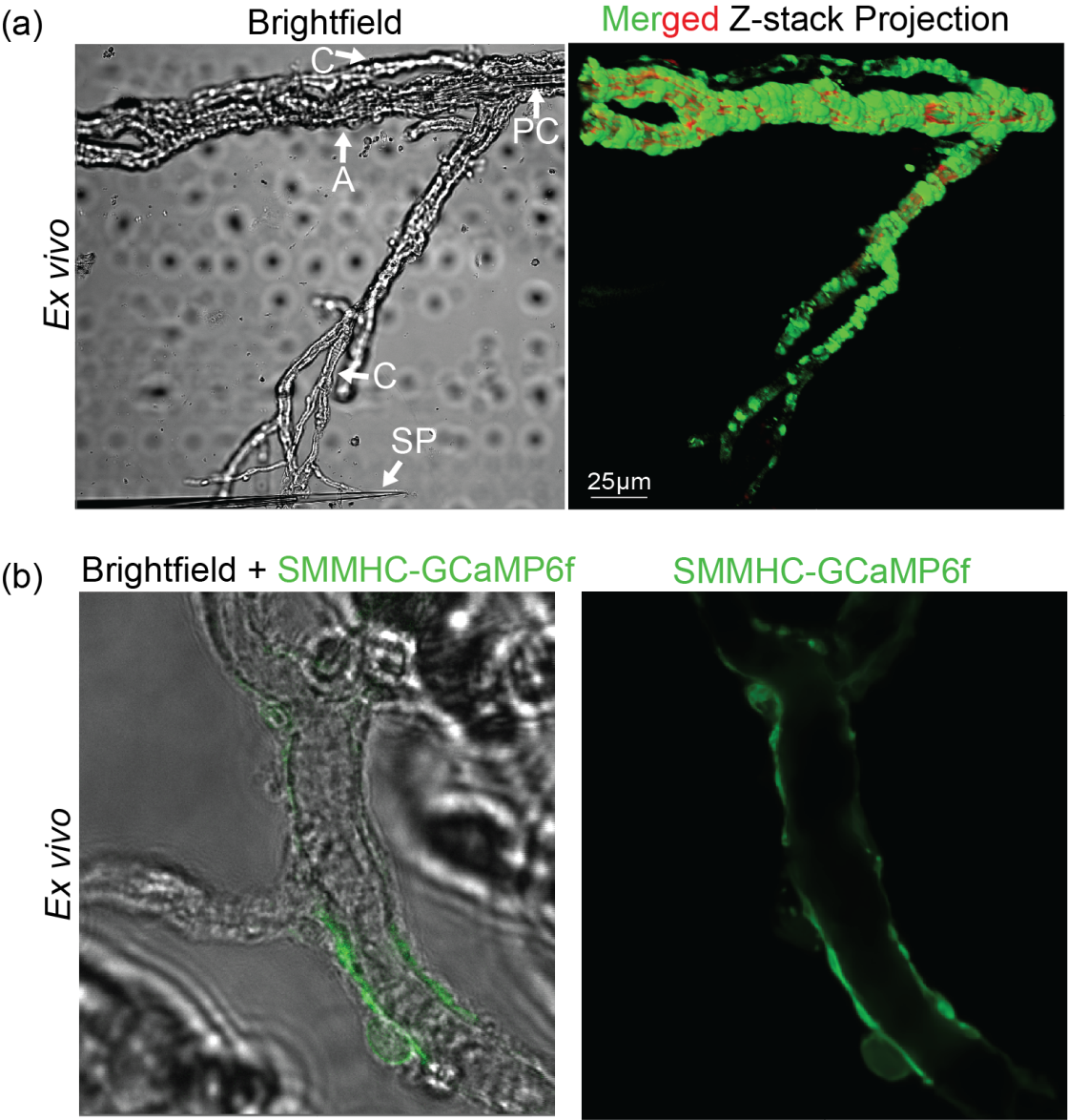


Supplemental Figure 1. Brightfield versus fluorescent channels. (a) Brightfield versus merged channels (SMMHC-GCaMP6f and Alexa Fluor™ 633) Z-stack projection of *ex vivo* CaPA preparation seen in Figure 2. Right panel is the same vessel image from 2d. Components are labeled, SP = Sealing Pipette, PC = Pressurizing Canula. Microvasculature is also indicated, C = Capillary, A = Arteriole, scale bar is shown. (b) Brightfield and SMMHC-GCaMP6f versus SMMHC-GCaMP6f – GFP channel of *ex vivo* CaPA preparation seen in Figure 5a and video Figure 5f.


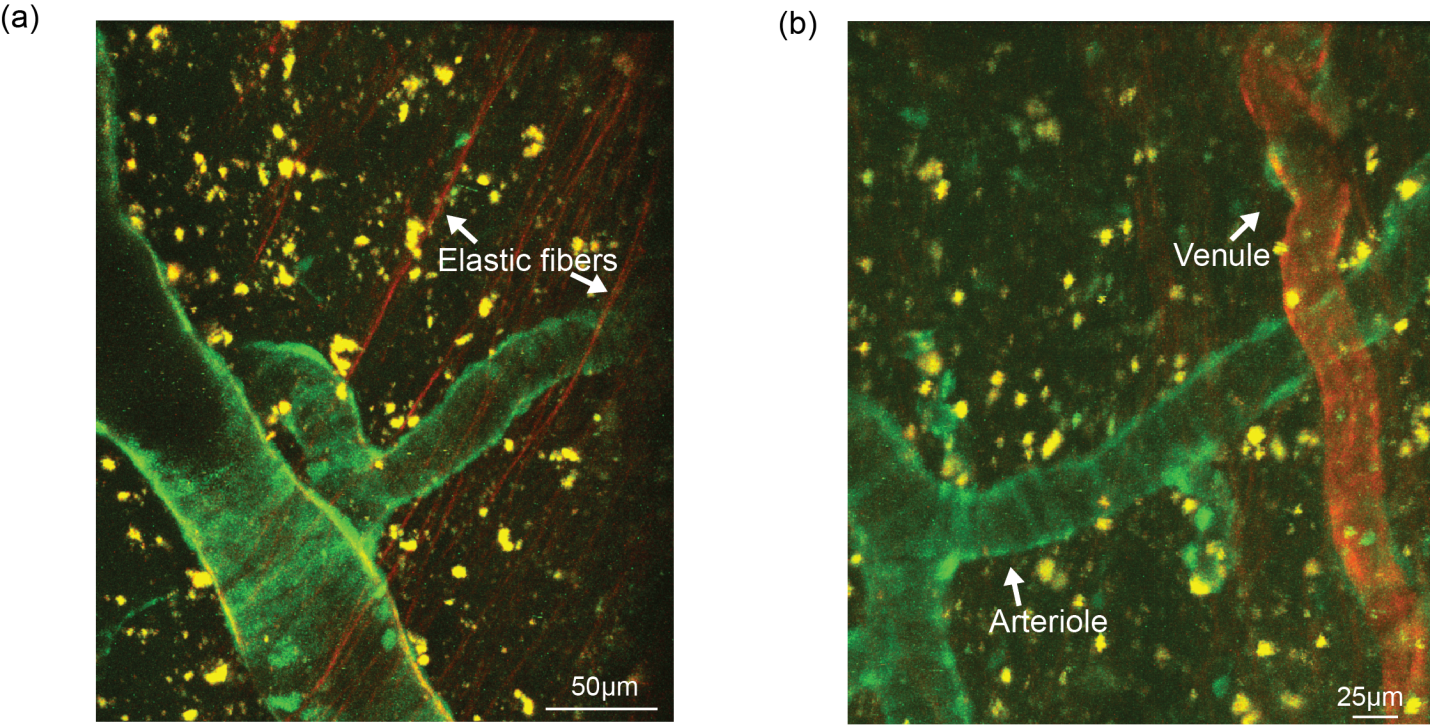


Supplemental Figure 2. *In vivo* elastic fiber and venule staining with Alexa Fluor™ 633 Hydrazide. (a) TPLSM imaging of cranial window post Alexa Fluor™ 633 Hydrazide retro orbital injection. Alexa Fluor™ 633 Hydrazide (red) was taken up by fibers throughout the cortex. Fibers are indicated by arrows and scale bar is shown. (b) TPLSM imaging of cranial window post Alexa Fluor™ 633 Hydrazide retro orbital injection from a different preparation. Veins took up the Alex Fluor™ 633 Hydrazide (red) more efficiently and red was present longer in this subset of microvasculature. Microvasculature is indicated by arrows and scale bar is shown.


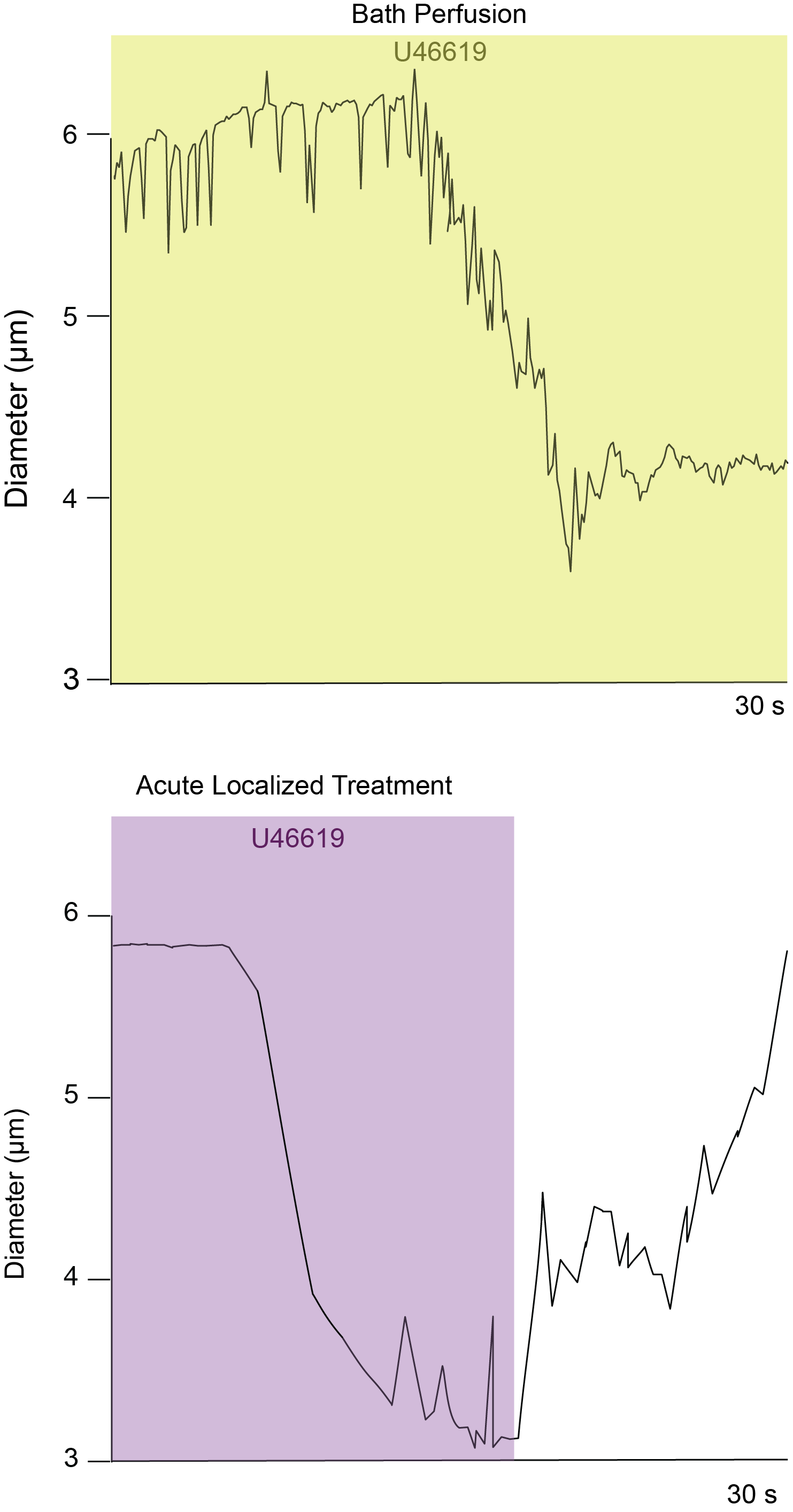


Supplemental Figure 3. *Ex vivo* CaPA representative traces of bath versus local application of U46619 zoomed-in from Figure 3c and 3d (second U46619 application). Consists of 30 seconds of continuous lumen diameter change with edge detection software (IonOptix). Exposure to 100 nM U46619 is shown by a colored rectangle.
